# Extracellular Regulation of Bone Morphogenetic Protein Activity by the Microfibril Component Fibrillin-1*

**DOI:** 10.1074/jbc.M115.704734

**Published:** 2016-04-08

**Authors:** Alexander P. Wohl, Helen Troilo, Richard F. Collins, Clair Baldock, Gerhard Sengle

**Affiliations:** From the ‡ Center for Biochemistry, Medical Faculty, University of Cologne, Joseph-Stelzmann-Street 52, 50931 Cologne, Germany,; the § Wellcome Trust Centre for Cell-Matrix Research and; ¶ Faculty of Life Sciences, University of Manchester, Michael Smith Building, Manchester M13 9PT, United Kingdom, and; the ‖ Center for Molecular Medicine Cologne (CMMC), University of Cologne, Robert-Koch-Street 21, 50931 Cologne, Germany

**Keywords:** bone morphogenetic protein (BMP), electron microscopy (EM), extracellular matrix, growth factor, signal transduction, small-angle X-ray scattering (SAXS), fribrillin

## Abstract

Since the discovery of bone morphogenetic proteins (BMPs) as pluripotent cytokines extractable from bone matrix, it has been speculated how targeting of BMPs to the extracellular matrix (ECM) modulates their bioavailability. Understanding these processes is crucial for elucidating pathomechanisms of connective tissue disorders characterized by ECM deficiency and growth factor dysregulation. Here, we provide evidence for a new BMP targeting and sequestration mechanism that is controlled by the ECM molecule fibrillin-1. We present the nanoscale structure of the BMP-7 prodomain-growth factor complex using electron microscopy, small angle x-ray scattering, and circular dichroism spectroscopy, showing that it assumes an open V-like structure when it is bioactive. However, upon binding to fibrillin-1, the BMP-7 complex is rendered into a closed ring shape, which also confers latency to the growth factor, as demonstrated by bioactivity measurements. BMP-7 prodomain variants were used to map the critical epitopes for prodomain-growth factor and prodomain-prodomain binding. Together, these data show that upon prodomain binding to fibrillin-1, the BMP-7 complex undergoes a conformational change, which denies access of BMP receptors to the growth factor.

## Introduction

Bone morphogenetic proteins (BMPs)^2^ belong to the TGF-β family of cytokines and play important roles during embryonic development and postnatal homeostasis of various organs and tissues, by controlling cellular differentiation, proliferation, and apoptosis (1, 2). These BMP-driven events underlie various control mechanisms in the extracellular space that can regulate the level, positioning, and timing of BMP signals (3). The most established mechanism of extracellular regulation of BMPs is represented by the concept of diffusible BMP antagonists that prevent access of BMPs to their signaling receptors and therefore effectively block BMP action (4). An alternative concept is that BMPs are targeted to extracellular scaffolds, such as fibrillin microfibrils (FMF) or collagen IV-rich networks that control and integrate BMP signaling in a context-specific manner (5, 6). FMF are 10–12-nm diameter extracellular scaffolds that are ubiquitously found in all tissues with a characteristic “beads on a string”-like appearance when extracted and visualized by negative-stained or rotary-shadowed electron micrographs. FMF serve as template for the deposition of elastin, but they are also found as non-elastin-associated independent networks (5, 7). Fibrillin deficiency leads to connective tissue disorders with opposing features characterized by tall *versus* short stature, hyperflexible *versus* stiff joints, hypo- *versus* hypermuscularity, and thin, hyperelastic, and translucent to thick, stiff, and hard skin (8). This clearly suggests that FMF control developmental and homeostatic events most likely by regulating the activity of connective tissue growth factors (9). Previously, we showed that targeting of BMPs to fibrillin-1 and fibrillin-2 is mediated through the prodomains (PDs) of BMPs (10, 11), which are generated by proteolytic cleavage from BMP precursors by the furin family of proprotein convertases before secretion. Two PDs associate in a non-covalent fashion with the mature growth factor (GF), the disulfide-cross-linked C-terminal cleavage product from the precursor. In contrast to PD-GF complexes of TGF-β-1, -2, and -3 (collectively called TGF-β), BMP complexes are not always latent. For example, it could be demonstrated that BMP-9 and BMP-7 are secreted by cells in the form of complexes that display the same bioactivity in solution as the mature GFs alone (12, 13). In-solution interaction assays with the BMP-7 complex and BMP receptors revealed a molecular mechanism through which type II receptors actively compete with the PD for the same binding epitope on the GF, resulting in displacement of the PD from the complex (13).

However, until now, it has not been known whether binding of BMPs through their PDs to fibrillin-1 confers latency to the GF or not and whether this interaction is part of a specific BMP sequestration mechanism mediated by the ECM. This information is highly relevant for understanding the underlying pathomechanisms of connective tissue disorders, such as Marfan syndrome, where an fibrillin-1-deficient matrix renders TGF-β PD-GF complexes unstable, resulting in aberrant TGF-β activation (14, 15). TGF-β is secreted in the form of large latent complexes, where the PD, also called latency-associated peptide (LAP), is tethered to latent TGF-β binding proteins (LTBPs) which target TGF-β to FMF via direct interaction of their C termini (16). BMPs bind directly to fibrillin-1 and -2, and BMP dysregulation due to fibrillin deficiency has been also observed in *Fbn1* and *Fbn2* knock-out mouse models (17–19). However, the molecular mechanisms by which this may occur remain obscure.

In this study, we determined for the first time the nanoscale structure of the BMP-7 complex, a PD-GF complex that is bioactive in solution, by using electron microscopy, small angle x-ray scattering (SAXS), and other biophysical approaches. We further mapped the binding site of the PD to the GF and to the PD itself and demonstrated that binding to fibrillin-1 confers latency to the GF induced through a conformational change of the complex. This suggests a new targeting and sequestration mechanism for BMPs induced by binding to the ECM.

## Experimental Procedures

### 

#### 

##### Protein Expression and Purification

**FIGURE 1.**
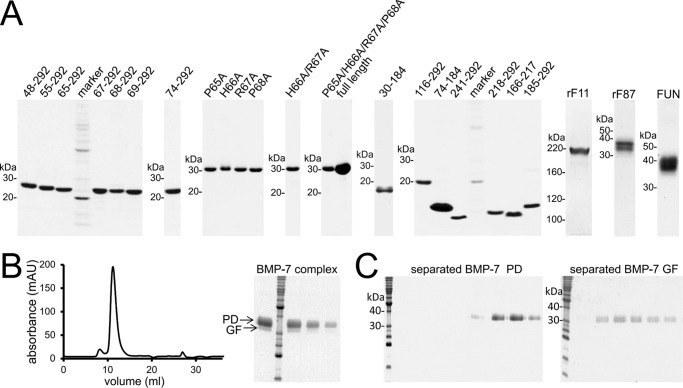
**Affinity purification of recombinant proteins used in this study.** *A*, Coomassie Brilliant Blue-stained SDS-polyacrylamide quality control gels of recombinantly expressed and affinity-purified BMP-7 PD variants and proteins representing the fibrillin-1 N terminus. *B* (*left*), size exclusion chromatogram of the BMP-7 PD-GF complex after chelating chromatography utilizing the His_6_ tag placed at the N terminus of the PD. The chromatogram shows the BMP-7 PD-GF complex mainly eluting in one peak. *Right*, Coomassie Brilliant Blue-stained SDS-polyacrylamide gel showing the purity of the peak fraction. *C*, Coomassie Brilliant Blue-stained SDS-polyacrylamide gels showing successful separation of the GF from the PD. The separation was performed as described previously (20). BMP-7 complex was separated into BMP-7 PD (34 kDa) and GF dimer (31 kDa) after dialysis into 8 m urea followed by chelating chromatography, where the PD was bound to the affinity column, and the GF was obtained in the flow-through.

A stably transfected BMP-7 complex expressing the HEK293 EBNA cell line and an expression plasmid coding for the N-terminal half of fibrillin-1 (rF11) were kindly provided by Dr. Lynn Sakai (Shriners Hospital for Children Portland, Oregon Health and Science University, Portland, OR) (20, 21). BMP-7 complex was purified by chelating chromatography utilizing a His_6_ tag placed at the N terminus of the PD followed by gel filtration. BMP-7 complex purification and separation into its components were as described previously (20). For separation of the GF from the PD, purified BMP-7 complex was dialyzed into 8 m urea, 1 m NaCl and subjected to a second chelating chromatography, where the PD was bound to the affinity column, and the GF was obtained in the flow-through (Fig. 1*B*). rF11 was overexpressed in HT1080 cells, followed by purification as described previously (21) (Fig. 1*A*). BMP-7 PD truncation variants were expressed in *Escherichia coli* with a C-terminal His_6_ tag and purified by chelating chromatography (10, 22) (Fig. 1*A*). BMP-7 PD point mutations were introduced using the QuikChange II site-directed mutagenesis kit (Agilent Technologies, Santa Clara, CA). rF87 (Ser^19^–Ile^329^) and the fibrillin-1 unique N-terminal domain (FUN) including the first EGF-like domain (Ser^19^–His^118^) containing the BMP-7 PD binding site fused together with cbEGF19-22 (Asp^1363^–Val^1527^) and a tandem C-terminal Strep-tag were expressed and purified as described previously (23) (Fig. 1*A*).

##### Transmission Electron Microscopy (TEM) and Single Particle Analysis

**FIGURE 2.**
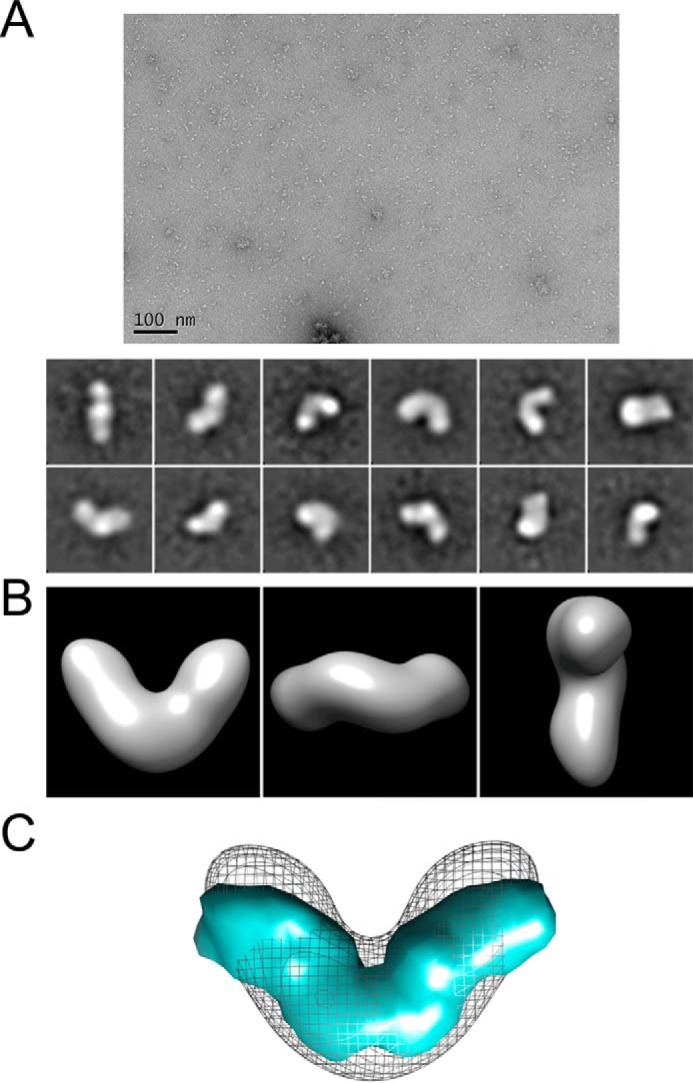
**Three-dimensional EM and solution SAXS models of BMP-7 PD-GF complex.** Three-dimensional structure of BMP-7 complex was generated using TEM. *A* (*top*), representative electron micrograph of BMP-7 complex molecules (*scale bar*, 100 nm); *bottom*, 12 images selected from 140 class sum images of 9,000 particles that represent different views of BMP-7 complex (*box size* = 29.4 × 29.4 nm). *B*, class sum images were used to generate a three-dimensional TEM model of BMP-7 complex with 2-fold symmetry using angular reconstitution. *C*, superimposition of the BMP-9 complex structure (33) at 20 Å with the determined BMP-7 EM envelope suggests that the angle between the boomerang arms may be wider in BMP-9.

BMP-7 complex alone or dialyzed together with the fibrillin-1 N terminus at a 1:4 molar ratio (total protein concentration 10–20 μg/ml) was negatively stained as described previously (24). Data were recorded at ×30,000 magnification on a Tecnai Biotwin microscope at 120 kV with a Gatan Orius CCD camera. Images were recorded with a 1-s exposure at defocus values of −0.5 to −1.6 μm at 3.5 Å/pixel (Fig. 2*A*). Single particle analysis was performed using EMAN2 (25). For BMP-7 complex, 9,000 particles were selected, and for BMP-7 dialyzed with fibrillin-1, 11,000 particles were selected, using a combination of manual and semiautomated picking. Following contrast transfer function correction, each data set was subjected to two-dimensional classification. For BMP-7 complex, a total of 140 projection averages were selected and used to generate an initial three-dimensional model with C2 symmetry. This model was used as a start seed for eight rounds of iterative refinement to produce a self-consistent three-dimensional structure. Resolution estimates were 35 Å using Fourier shell correlation with a cut-off value of 0.5. Modeling was performed using UCSF Chimera (26).

##### SAXS Data Collection and Analysis

SAXS intensity data on the BMP-7 complex were collected at 10 °C using a BioSAXS robot at the EMBL-P12 beamline at PETRAIII (Deutsche Elektronen Synchrotron (DESY), Hamburg) and the ESRF on BM29. The scattering images were obtained using automated data acquisition and radially averaged using in-house software. The data were merged to cover a *q*-range of 0.008 < *q* < 0.54 Å^−1^ from a concentration range 1–5 mg/ml. For all SAXS data, the *R_g_* and distance distribution function *p*(*r*) were evaluated with GNOM (27). Particle shapes were generated *ab initio* using DAMMIN or GASBOR software with 2-fold symmetry (27).

##### Circular Dichroism Spectroscopy

BMP-7 PD variants were dialyzed in 5 mm HClO_4_ and concentrated in Amicon Ultra 0.5-ml centrifugal filters (Millipore) to 0.2–1 mg/ml. CD spectra were recorded using a Jasco J-715 spectropolarimeter at 260 to 170 nm in a 0.1-mm path length quartz cell (Hellma, Germany) at 20 °C. After subtraction of the buffer contribution, data were converted to Δϵ. Percent secondary structure was calculated with the online server DICHROWEB (28, 29) using the SELCON (30, 31) and CDSSTR algorithms (31).

##### ELISA-style Binding Assays

Binding assays were performed as described previously (13). BMP-7 PD variants were coated at 0.1 μm to Nunc MaxiSorp flat-bottom 96-well plates (Thermo Scientific, Waltham, MA), and BMP-7 GF was incubated in solution. Bound GF was detected using a polyclonal anti-BMP-7 GF antibody (500-P198, Peprotech, Rocky Hill, NJ). Sandwich ELISAs were performed by coating a monoclonal anti-His_6_ tag antibody (clone AD1.1.10, R&D Systems, Minneapolis, MN) at 1 μg/ml. BMP-7 PD variants were dialyzed together with the GF in TBS containing 1 m urea overnight followed by incubation with the coated capture antibody and detection of the GF.

##### Surface Plasmon Resonance

SPR experiments were performed as described previously (10, 13) using a BIAcore 2000 system (BIAcore AB, Uppsala, Sweden). BMP-7 GF was covalently coupled to CM5 sensor chips at 500 resonance units (RUs), and PD variants were flown over in HBS-EP buffer (0.01 m HEPES, pH 7.4, 0.15 m NaCl, 3 mm EDTA, 0.005% (v/v) surfactant P20) (BIAcore AB) containing 1 m urea. To test the PD/GF interaction at pH 4.5, 1,000 RUs of BMP-7 PD were immobilized, and 0–80 nm GF was injected in 10 mm sodium acetate buffer, pH 4.5, 0.15 m NaCl, 3 mm EDTA, 0.005% (v/v) surfactant P20. For PD/PD interaction experiments, 500–800 RUs of full-length PD (residues 30–292) or truncated variants (residues 48–292 or 55–292) were immobilized on the chip, and potential self-interaction was tested by injecting 0–320 nm PD variants in HBS-EP, 1 m urea. For interaction experiments with rF87 and BMP-7 PD truncation variants, PD variants were immobilized at 500–800 RUs, and rF87 was injected at 0–80 nm in HBS-EP. For competition experiments with the BMP type II receptor, PD variants were immobilized at 500–800 RUs, and 100 nm BMP-7 GF was injected in the presence of 0–500 nm of soluble extracellular domain of BMPRII receptor (R&D Systems) followed by a subsequent 100 nm injection (“coinject” mode) of a monoclonal anti-BMP-7 GF antibody (MAB3542, R&D Systems) to detect bound GF. All injections were in HBS-EP buffer. Kinetic constants were calculated by nonlinear fitting (1:1 interaction model with mass transfer) to the association and dissociation curves according to the manufacturer's instructions (BIAevaluation version 3.0 software). Apparent equilibrium dissociation constants (*K_D_* values) were then calculated as the ratio of *k_d_*/*k_a_*.

##### Velocity Sedimentation

10 μg of BMP-7 complex dialyzed against TBS containing 0.25–4 m urea was applied to a 5–20% (w/v) sucrose gradient. Ultracentrifugation was carried out as described previously using a Beckman L8-M ultracentrifuge (13). Each gradient was collected in 28 fractions and analyzed by Western blotting using specific anti-BMP-7 PD (mAb2 from Millipore, Billerica, MA) and GF (500-P198, Peprotech) antibodies (13).

##### Combined ELISA/BMP Bioactivity Assay

mAb anti-His_6_ (clone AD1.1.10, R&D Systems) and rF11 (both at 10 μg/ml) were adsorbed overnight to 96-well ELISA plates (Nunc). 5% milk was incubated as a blocking solution for 1 h, followed by three TBS/Tween washes and subsequent incubation with 0.5 μm BMP-7 complex in 2% milk for 2 h. To quantify the bound amounts of BMP-7 complex, the plate was incubated with stripping solution (100 μl/well of 0.1 m glycine, pH 2.3) for 20 min. Each time, the contents of 96 wells were pooled and dialyzed against 0.1 m acetic acid overnight. This solution was lyophilized and resuspended in 50 μl of TBS. 5-μl dots were placed on a nitrocellulose membrane together with a diluted series of dots containing BMP-7 complex at known concentrations serving as a standard curve. After drying, the membrane was blocked in 5% milk and incubated with anti-BMP-7 PD antibody mAb2 (Millipore) (1 mg/ml, 1:500) for 2 h. The membrane was washed and subsequently incubated with an HRP-conjugated goat anti-mouse antibody for 2 h. After the final washes, signals were developed using the Bio-Rad Opti 4CN Substrate kit. The membrane was scanned, and signals were quantitated using ImageQuant. C2C12 cells were seeded onto plates in which quantitated amounts of BMP-7 complex had been immobilized. After 5 h, the total mRNA was immediately harvested, and mRNA expression levels of *Id3*, a BMP-responsive element, were monitored by real-time quantitative PCR. BMP-7 complex was added at 300 ng/ml to the medium as positive control, and BSA-coated wells served as negative control.

##### Homology Modeling

The BMP-7 PD was modeled, using the TGF-β-1 LAP crystal structure (Protein Data Bank code 3RJR) as a template, with MODELLER (32) in UCSF Chimera (26). A sequence alignment, generated partially with ClustalW with some manual realignment guided by CD secondary structural information, was inputted to guide homology modeling. In LAP, the first 28 amino acids form the α1-helix, but because our CD shows that the first 12 residues of BMP-7 PD are not helical, these amino acids were refined with MODELLER, and an unstructured conformation that allowed interaction between the BMP-7 PDs was selected. The structure of the BMP-7 GF (Protein Data Bank code 1BMP) was substituted for TGF-β-1 GF. A break in the peptide chain was introduced at residue Pro^80^ to allow the PD to be rotated into an open conformation without moving the N-terminal region. The BMP-9 complex structure (Protein Data Bank code 4YCI) and the BMP-7 complex EM map were used to guide rotation of the PD into an open conformation.

## Results

### 

#### 

##### Nanoscale Structure of the BMP-7 Complex

**FIGURE 3.**
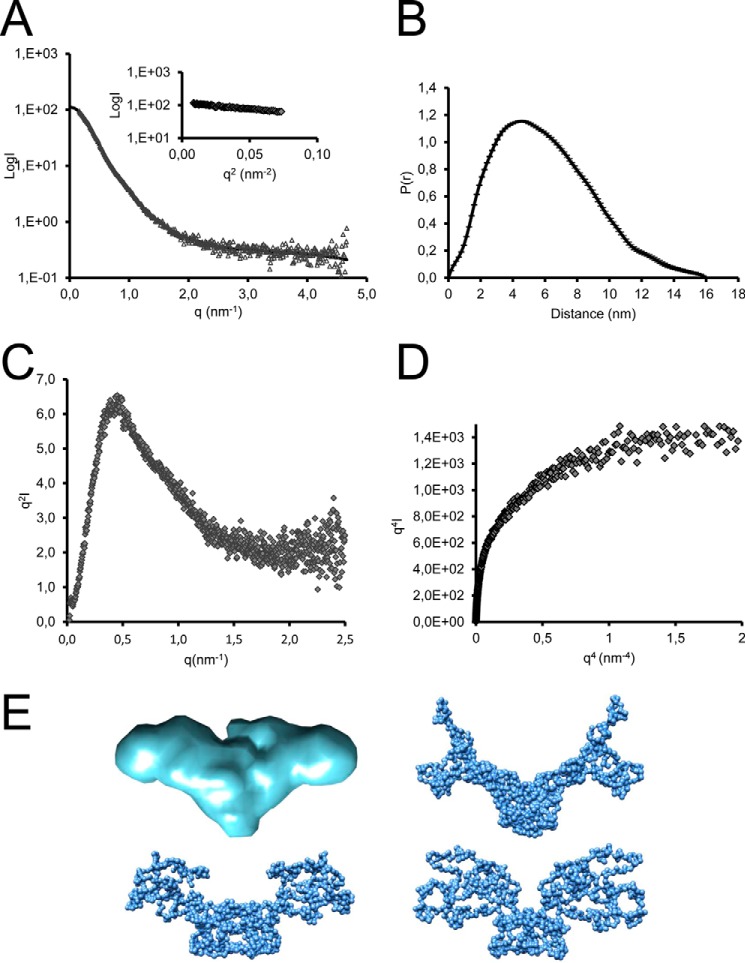
**SAXS data collected for BMP-7 complex.** *A*, x-ray scattering profile of BMP-7 complex showing intensity as a function of *q* (*gray triangles*) and Gnom fit to the data (*black line*). *Inset*, Guinier plot showing *R_g_* of 4.8 nm. *B*, *P*(*r*) plot showing *D*_max_ of 16 nm. *C*, Kratky plot showing profile typical of a folded protein. *D*, plateau in the Porod-Debye plot indicative of a non-flexible protein. *E*, *ab initio* models generated from SAXS data using GASBOR with 2-fold symmetry (*blue*); the averaged model is shown along with three representative models.

To determine the shape and structure of the BMP-7 complex, we used single-particle TEM. Recombinantly expressed and affinity-purified BMP-7 complex (Fig. 1*B*) was negatively stained (Fig. 2*A*), and particles were classified using reference-free alignment (Fig. 2*A*) and used to calculate a three-dimensional reconstruction with 2-fold symmetry to a 35 Å resolution. The three-dimensional structure of BMP-7 complex reveals an open boomerang-shaped conformation with dimensions 13 × 9 × 5 nm (Fig. 2*B*) similar to the BMP-9 complex structure (33). However, superimposition of the BMP-9 complex at 20 Å resolution with the determined BMP-7 EM envelope suggests that the angle between the boomerang arms may be wider in BMP-9 (Fig. 2*C*). Independently, we determined the three-dimensional shape of the BMP-7 complex in solution using SAXS. Measurements were made at the DESY and European Synchrotron Radiation Facility (ESRF) (Fig. 3). The data quality was assessed using a Guinier plot, and the *R_g_* obtained was 4.8 nm (Fig. 3*A*). *Ab initio* bead models were generated using the program GASBOR with 2-fold symmetry. At least 20 separate simulations were completed to determine the common structural features; the models fit the experimental data with a mean discrepancy factor χ of 0.96, and variation between the models had a mean normalized spatial discrepancy factor of 2.25. The averaged model had dimensions of 15 × 8 × 5 nm (Fig. 3*E*). The overall shape of the SAXS model is similar to that determined by EM, and together they show that the BMP-7 complex has an open conformation similar to the recently determined BMP-9 complex structure (33) rather than a closed ring-shaped structure like the SLC of TGF-β-1 (34).

##### Secondary Structure Analysis of the BMP-7 Prodomain

**FIGURE 4.**
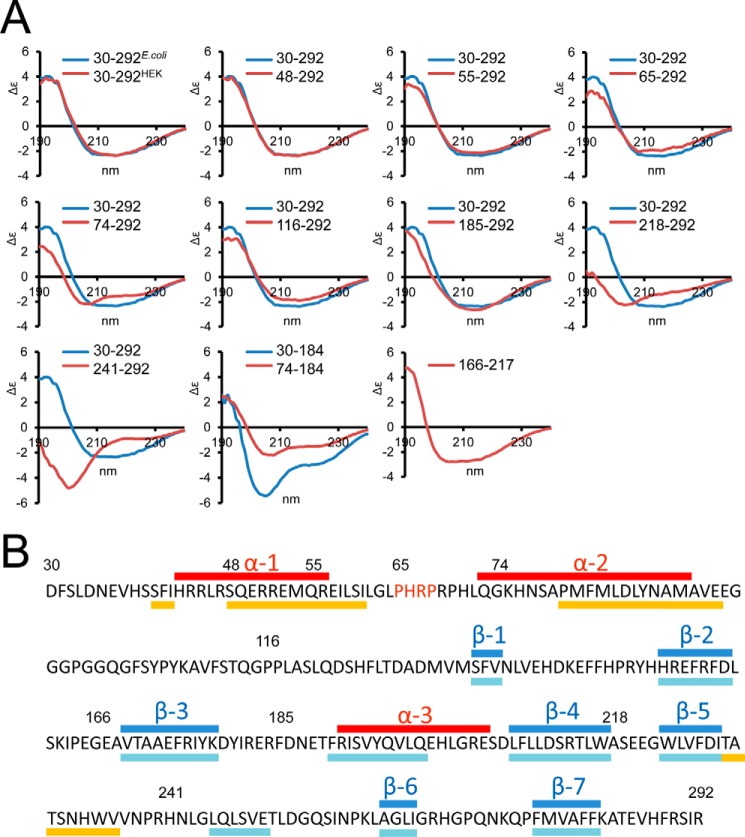
**Secondary structure analysis of BMP-7 PD.** *A*, CD measurements of N-terminal BMP-7 PD truncation variants (*red*) in comparison with full-length BMP-7 PD (30–292, *blue*). *Bottom panel*, *middle*, truncation of the first 43 residues in a shorter variant covering the first 184 N-terminal residues (*red*) results in significant reduction of the α-helical peak at 209 nm compared with the control (*blue*). Construct 166–217 suggests the existence of a third α-helix within this region. Secondary structure percentage calculated from these CD curves is listed in Tables 1 and 2. *B*, secondary structure prediction based on CD measurements shows the position of α-helices (*red*). The position of β-sheets (*blue*) was guided by predicted secondary structure elements (PSIPRED) marked *below* the sequence (*yellow*, α-helical regions; *light blue*, β-sheets).

Based on sequence homology to the TGF-β-1 PD, it was previously predicted that the N-terminal region of the BMP-7 PD forms an amphipathic α-helix containing hydrophobic residues that mediate specific interactions with the mature GF (34, 35). Interestingly, a similar N-terminal α1-helix was also predicted for the BMP-9 PD (33); however, the BMP-9 complex structure revealed that the C-terminal α5-helix represents the main binding interface with the GF (33). Although predicted using the PSIPRED server (Fig. 4*B*), we wanted to confirm the existence of a similar N-terminal α-helical region within the BMP-7 PD by using CD spectroscopy. Previous CD studies have shown that the α-helical content of the BMP-7 PD remains unaltered after separation from the GF (20). We therefore determined the overall location of α-helical regions by generating and analyzing a systematic set of truncation variants spanning full-length BMP-7 PD. All proteins were expressed in *E. coli* and purified to >95% purity as assessed by SDS-PAGE followed by Coomassie staining (Fig. 1*A*). CD spectra from full-length BMP-7 PD expressed in *E. coli* and separated PD from complex obtained from mammalian cell culture showed perfect overlay (Fig. 4*A*), indicating that carbohydrate chains absent in the *E. coli* preparation have no influence on PD secondary structure. Determining the difference in measured α-helical content in a series of fragments with systematic N-terminal truncations and also in smaller fragments spanning the entire PD sequence allowed us to predict a secondary structure map, which suggests the presence of two α-helical regions (α1 (His^43^–Arg^56^) and α2 (Gln^73^–Met^90^)) within the first 86 amino acids following the signal peptide cleavage site (Fig. 4*B* and Tables 1 and 2). With the exception of a third α-helix (α3, Arg^191^–Glu^205^), we found that the region after Gly^97^ contains mostly β-strands (Fig. 4*B* and Tables 1 and 2).

**TABLE 1:** **Percent secondary structure of BMP-7 PD variants** Secondary structure percentage (with the corresponding number of residues in parentheses) was calculated from CD curves shown in Figs. 4*A* and 5*E* using the online server DICHROWEB (28, 29), employing the SELCON (30, 31) and CDSSTR (31) algorithms. All point mutations were introduced within the context of full-length BMP-7 PD (residues 30–292).

BMP-7 PD variant	α-Helix	β-Sheet	Turns	Unordered	Total
30–292 from 293 cells	18 (47)	33 (87)	12 (32)	37 (97)	100 (263)
30–292	19 (50)	32 (84)	12 (32)	37 (97)	100 (263)
48–292	18 (44)	33 (81)	12 (29)	37 (91)	100 (245)
55–292	16 (38)	34 (81)	12 (29)	38 (90)	100 (238)
65–292	16 (36)	34 (78)	12 (27)	38 (87)	100 (228)
74–292	16 (35)	31 (68)	14 (30)	39 (85)	100 (218)
116–292	10 (18)	39 (69)	12 (21)	39 (69)	100 (177)
30–184	18 (28)	21 (33)	22 (34)	39 (60)	100 (155)
74–184	15 (17)	32 (36)	12 (13)	41 (45)	100 (110)
185–292	16 (17)	27 (29)	14 (15)	43 (47)	100 (108)
166–217	32 (16)	26 (13)	11 (5)	31 (16)	100 (51)
218–292	5 (4)	26 (19)	19 (14)	50 (37)	100 (74)
241–292	3 (2)	21 (11)	19 (10)	57 (30)	100 (52)
67–292	24 (53)	27 (62)	12 (27)	37 (84)	100 (226)
68–292	21 (48)	30 (67)	12 (27)	37 (83)	100 (225)
69–292	17 (39)	34 (75)	11 (26)	38 (84)	100 (224)
P65A	23 (60)	27 (71)	13 (34)	37 (97)	100 (263)
H66A	19 (50)	32 (84)	12 (32)	37 (97)	100 (263)
R67A	18 (47)	35 (92)	12 (32)	35 (92)	100 (263)
P68A	19 (50)	29 (76)	13 (34)	39 (103)	100 (263)
H66A/R67A	18 (47)	31 (82)	13 (34)	38 (100)	100 (263)
P65A/H66A/R67A/P68A	23 (60)	31 (82)	11 (29)	35 (92)	100 (262)

**TABLE 2:**
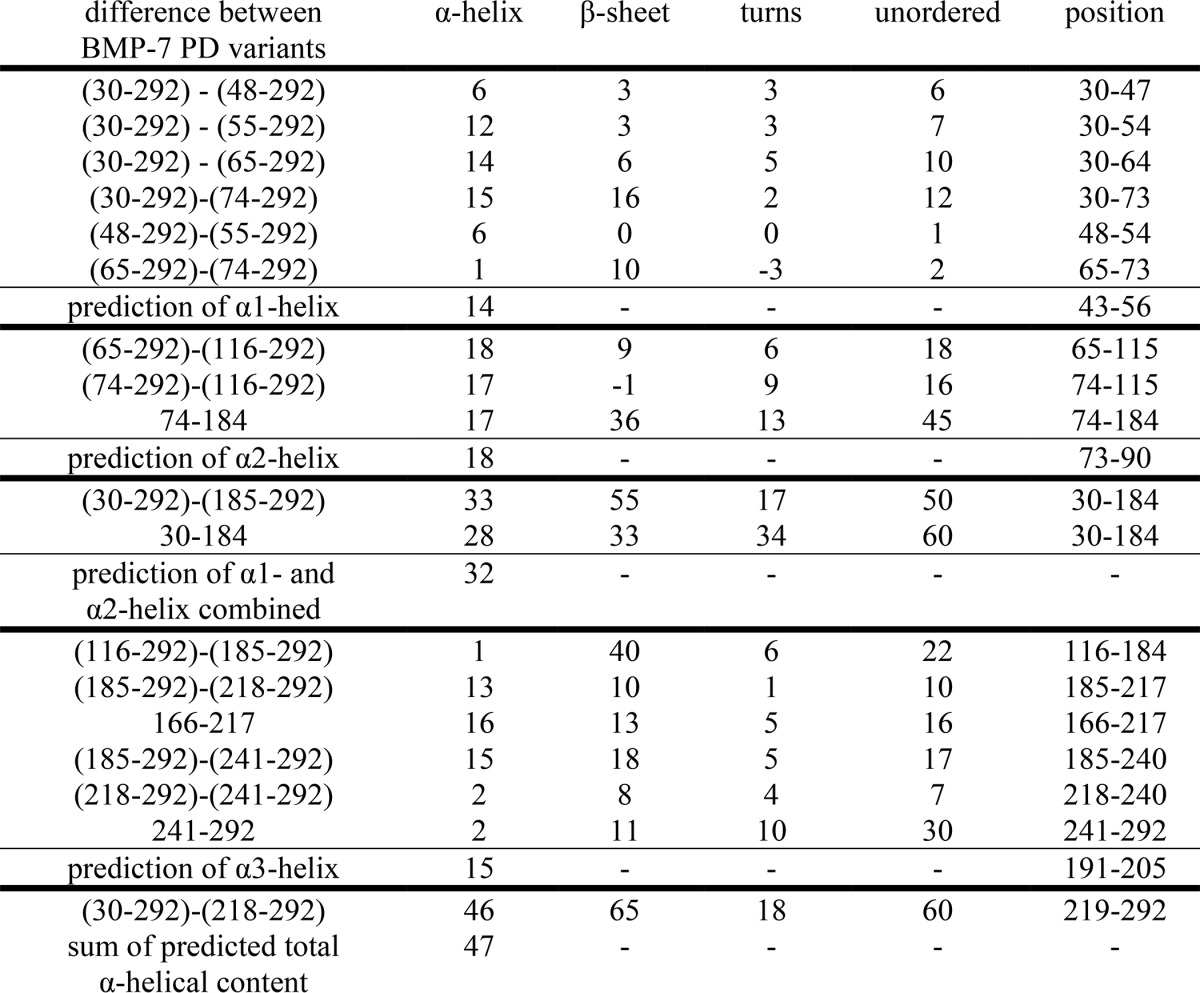
**Difference of measured α-helical content in truncation variants predicts the presence and localization of three α-helical regions within the BMP-7 PD** Numbers represent number of residues.

##### Determination of the Growth Factor Binding Motif within the BMP-7 Prodomain

**FIGURE 5.**
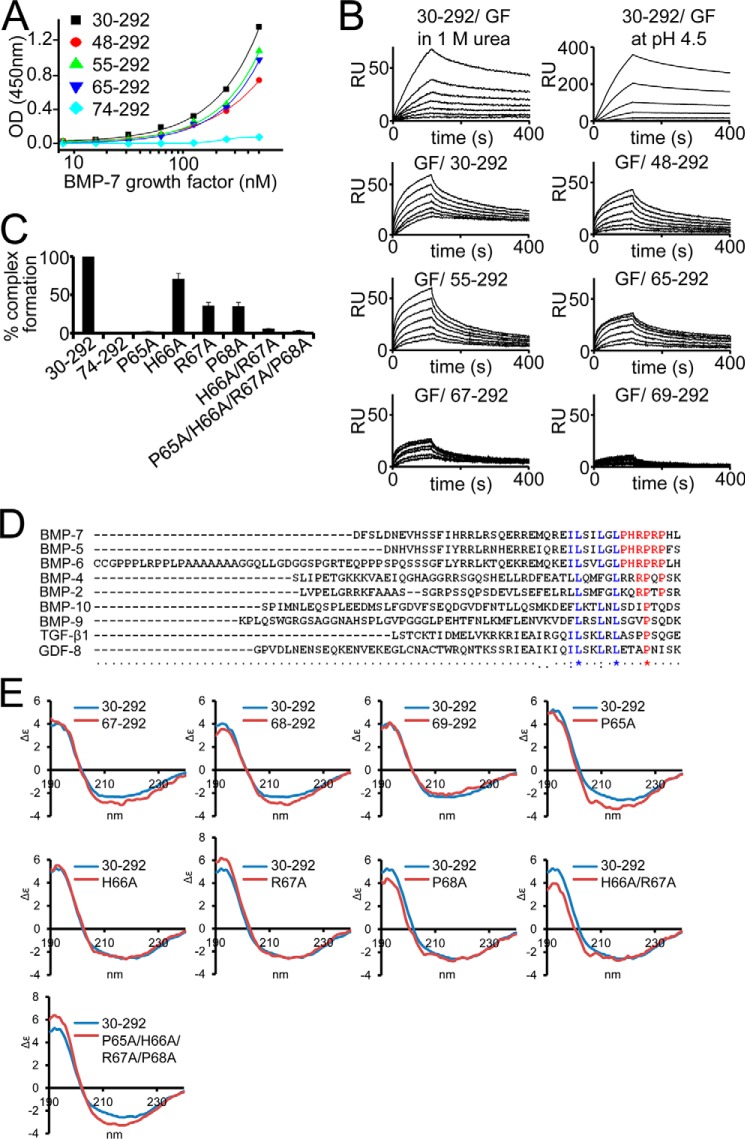
**Identification of the GF binding motif within the BMP-7 PD.** *A*, solid phase binding ELISA-style assays with immobilized BMP-7 PD truncation variants and GF in solution. *B*, SPR binding studies of BMP-7 N-terminal PD truncation variants and BMP-7 GF. *Top panel*, GF binding to the PD was robust to the presence of 1 m urea and pH reduction to 4.5 (full-length PD(30–292) immobilized, GF in solution). *Bottom panels*, GF was immobilized, and PD variants were injected in solution. *C*, BMP-7 reconstitution after dialysis of PD variants and GF. Successful reconstitution was monitored in a sandwich ELISA using an anti-His_6_ antibody against the C-terminal His_6_ tag on the PD as capture and a polyclonal anti-BMP-7 GF antibody as detector. *Error bars*, S.D. from three independent experiments. *D*, sequence alignment using ClustalW identifies the ^65^PHRP^68^ motif (*red*) as conserved within the BMP-5, -6, -7 subgroup of the TGF-β superfamily. *Blue*, predicted Ile^58^-Leu^59^-Leu^62^-Leu^64^ GF binding motif (35). *E*, CD spectra of systematic truncation variants between Arg^67^ and Pro^69^. Deletion of Pro^65^-His^66^ results in a significant increase of α-helical content of 8% when compared with the 65–292 PD variant (Table 1). This increase returned to normal levels upon stepwise N-terminal truncation of the subsequent two residues. The point mutation P65A resulted in a 4% increase of α-helical content (Table 1), whereas H66A or P68A resulted in no or little change. Additional mutation of the subsequent three residues resulted in no additional change in α-helical content in the quadruple mutant variant P65A/H66A/R67A/P68A (Table 1).

We wanted to identify the critical residues of the GF binding motif within the BMP-7 PD. To investigate whether the N-terminal region is important for GF binding, as previously predicted (34, 35), we subjected our set of systematic N-terminal truncation variants of the BMP-7 PD to an interaction screen with the GF using ELISA-style binding assays (Fig. 5*A*). In this assay, the PD was immobilized, and the GF was incubated in solution. We found that upon deletion of the first 44 residues after the signal peptide cleavage site, binding was completely abolished. This suggests that the GF binding site is fully contained within this region. However, deletion of the first 35 residues containing the predicted Ile^58^-Leu^59^-Leu^62^-Leu^64^ motif (35) diminished GF binding by 30% (Fig. 5*A*), indicating that the important residues of the GF binding motif are contained within a stretch of nine amino acid residues: ^65^PHRPRPHLQ^73^.

To verify this observation, we wanted to perform binding studies in the opposite direction (GF immobilized on a chip, PD variants in solution) employing surface plasmon resonance (SPR). In previous SPR BMP-7 PD/GF interaction studies (PD immobilized, GF in solution), we had determined a *K_D_* of 21 nm when the separated components from the HEK293 EBNA cell-expressed complex were used in normal HEPES buffer (HBS-EP) without urea (13). We found that changing the direction of the assay required the addition of 1 m urea in the running buffer to overcome solubility problems with the PD variants. We first monitored any change in affinity when *E. coli*-derived full-length PD (residues 30–292) was immobilized and the GF was flown over in the presence of 1 m urea. In this set-up, the PD/GF affinity proved to be fairly robust because it only dropped slightly to 31 nm. Also, a pH reduction to 4.5 had no inhibitory effect (Fig. 5*B* and Table 3), showing the robustness of this interaction. Changing the direction of the assay in the presence of 1 m urea did not negatively influence the GF/PD interaction because the affinity was determined to be 19 nm (Fig. 5*B* and Table 3). By performing GF/PD SPR interaction studies, we confirmed that only deletion of ^65^PHRPRPHLQ^73^ completely abolished GF binding (Fig. 5*B* and Table 3). SPR interaction experiments with PD variants covering regions C-terminal to ^65^PHRPRPHLQ^73^, such as Pro^116^–Arg^292^, Gly^166^–Arg^217^, or Phe^185^-Arg^292^, showed no binding, which further corroborates the observation that the GF binding site resides within the N-terminal region (Table 3). To further narrow down the GF binding site, we generated additional truncation variants within the presumptive binding epitope ^65^PHRPRPHLQ^73^. Further deletion of the next succeeding four residues completely ablated binding to the GF, suggesting that the region ^65^PHRP^68^ is essential for maintaining GF binding (Fig. 5*B* and Table 3).

**TABLE 3:** **Dissociation constants (*K_D_*) determined by fitting surface plasmon resonance curves obtained for the performed interaction experiments** Ligands were immobilized on sensor chips and analytes were flown over in HBS-EP (0.01 m HEPES, pH 7.4, 0.15 m NaCl, 3 mm EDTA, 0.005% (v/v) surfactant P20) plus 1 m urea except where noted. Data represent the average of three independent experiments. NM, not measurable.

Ligand/Analyte	*K_D_*
	*nm*
GF/30–292	19.4 ± 1 1.04
GF/48–292	19 ± 1
GF/55–292	15 ± 3
GF/65–292	8 ± 1
GF/74–292	NM
GF/67–292	NM
GF/69–292	NM
GF/116–292	NM
GF/166–217	NM
GF/185–292	NM
30–292/GF	31.5 ± 0.2
30–292/GF	20 ± 0.1*^a^*
30–292/30–292	3 ± 0.01
48–292/48–292	NM
55–292/55–292	NM
30–292/74–292	NM
30–292/166–217	NM
30–292/185–292	NM
30–292/218–292	26.8 ± 3.1
30–292/241–292	39.1 ± 3.3
30–292/fibrillin-1 (rF87)	32 ± 7*^b^*
30–217/fibrillin-1 (rF87)	27 ± 6*^b^*
30–184/fibrillin-1 (rF87)	38 ± 3*^b^*
74–292/fibrillin-1 (rF87)	26 ± 4*^b^*

*^a^* 20 mm sodium acetate, pH 4.5, 0.15 m NaCl, 3 mm EDTA, 0.005% (v/v) surfactant P20.

*^b^* HBS-EP only.

Sequence alignment of PDs of the TGF-β superfamily members known to form active or latent PD-GF complexes showed that the identified motif is fully conserved within the BMP-5, -6, -7 subgroup, in which at least BMP-5 and -7 were shown to assemble stable and bioactive complexes (10, 11) (Fig. 5*D*). To investigate whether the ^65^PHRP^68^ motif is also required for complex formation and stability in solution, we generated point mutations within this motif and dialyzed the resulting PD variants together with the GF dimer to reconstitute the BMP-7 complex as shown previously (10, 11). Successfully formed BMP-7 complexes were monitored by sandwich ELISA using a monoclonal anti-His_6_ antibody against the PD C terminus as capture and an anti-GF antibody as detector. Simultaneous mutation of all residues to alanine (P65A/H66A/R67A/P68A) resulted in complete inhibition of BMP-7 complex formation. Complete inhibition of complex formation could also be achieved by simultaneous double mutation of H66A/R67A. Individual single mutations had different impact on complex formation. H66A resulted in 30% inhibition, R67A or P68A resulted in about 65% inhibition, and P65A completely inhibited complex formation (Fig. 5*C*). The strong inhibitory effect of P65A may be reflected by the change of secondary structure induced by this mutation. P65A resulted in a 4% increase in α-helix (conversion of an additional 10 residues), whereas H66A or P68A resulted in no or little change in secondary structure content (Fig. 5*E* and Table 1). Also, additional mutation of the subsequent three residues resulted in no additional change in α-helical content in the quadruple mutant variant P65A/H66A/R67A/P68A (Fig. 5*E* and Table 1). Furthermore, truncation of Pro^65^-His^66^ resulted in a significant increase of α-helical content of 8% (corresponding to 17 residues of additional α-helical content) when compared with the 65–292 PD variant (Fig. 5*E* and Table 1). Together, these data suggest that Pro^65^ is important for maintaining secondary structure elements within the subsequent PD sequence.

##### The BMP-7 Prodomain Interacts with Itself

**FIGURE 6.**
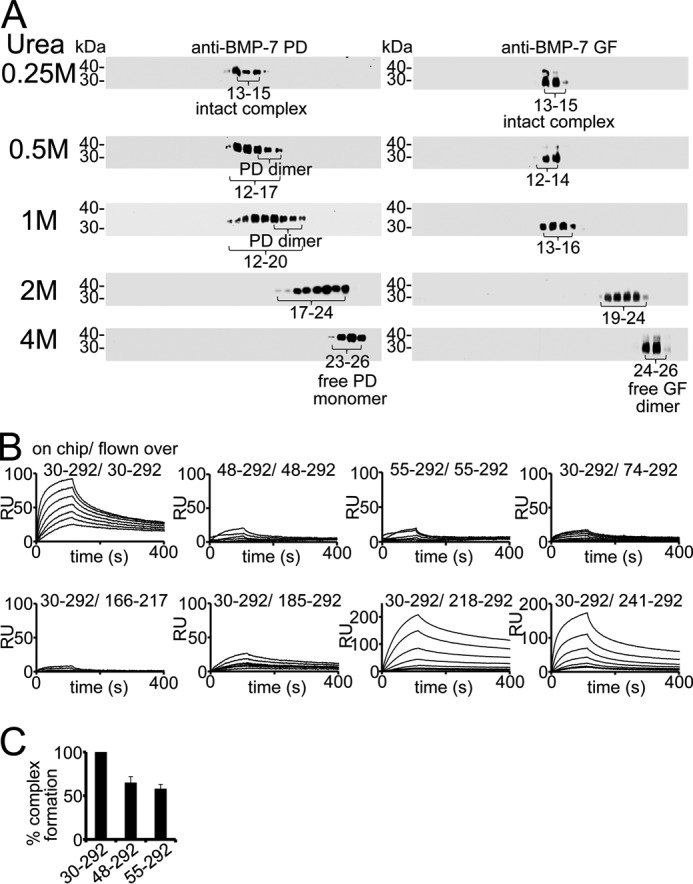
**BMP-7 PD interacts with itself.** *A*, dialysis of BMP-7 complex into 0.25–4 m urea reveals the presence of PD dimers monitored by velocity sedimentation experiments using 5–20% sucrose gradients. Each gradient was collected in 28 fractions (fraction 1 at 5% and fraction 28 at 20% sucrose) and subjected to Western blotting analysis for BMP-7 complex components. Western blots were incubated with anti-BMP-7 PD antibody first, stripped, and subsequently re-incubated with anti-BMP-7 GF antibody. *B*, SPR sensorgrams of self-interaction studies with BMP-7 full-length PD, 48–292, and 55–292, respectively, of immobilized full-length PD and PD variants representing the C-terminal end. *C*, BMP-7 complex reconstitution is affected by 30% upon deletion of the N-terminal PD self-interaction site. *Error bars*, mean ± S.D. from three independent experiments.

Because the BMP-7 complex assumes an open V-shape in solution, we were interested in whether this conformation allows PD-PD contact. Previous sedimentation velocity experiments suggested that upon type II receptor binding, the PD is released as a dimer, suggesting the possibility of PD self-interaction (13). To analyze the presence of PD dimers in the BMP-7 complex, we performed a velocity sedimentation experiment, where we monitored the successive separation of the complex into its components upon dialysis into increasing amounts of urea (0.25–4 m) (Fig. 6*A*) to reveal the presence of PD dimers as intermediates in the stepwise dissociation process. In the complexed state, the signals for both BMP-7 components were detected in the same position in the gradient (fractions 13–15 at 0.25 m urea). However, upon the addition of 0.5 m urea, the complex starts to slightly unfold, which results in the presence of additional PD signals in fractions not containing the GF (fractions 15–17 at 0.5 m urea). At 1 m urea, the complex unfolded further, revealing the presence of additional PD-only signals (fractions 17–20), which are shifted more than five fractions up compared with the position of completely separated monomers (fractions 23–26 at 4 m urea) (Fig. 6*A*). These PD-only separation intermediates disappeared in the presence of higher urea concentrations, suggesting that the PD is present as a dimer within the assembly of the BMP-7 PD-GF complex.

To verify this observation, we performed self-interaction studies with the BMP-7 PD employing SPR. Our results indicated that the BMP-7 PD interacts with itself with a molecular affinity of 3 nm (Table 3). To localize the self-interaction motif, we carried out a self-interaction screen and found that truncation of the first 18 N-terminal residues abolished PD self-interaction (Fig. 6*B*). Interestingly, although full-length BMP-7 PD and N-terminal truncation variants did not interact, shorter variants representing the C-terminal region (218–292 and 241–292) showed binding (Fig. 6*B* and Table 3). This suggests that this C-terminal self-interaction motif located within Ala^218^-Arg^292^ is cryptic within the full-length BMP-7 PD.

To evaluate the importance of the N-terminal self-interaction motif for complex formation and stability, we again carried out in-solution reconstitution assays with the GF. Upon truncation of the self-interaction motif, complex formation was inhibited by 35%, suggesting that PD-PD interactions contribute to overall complex stability (Fig. 6*C*).

##### Competition with the BMP Type II Receptor

**FIGURE 7.**
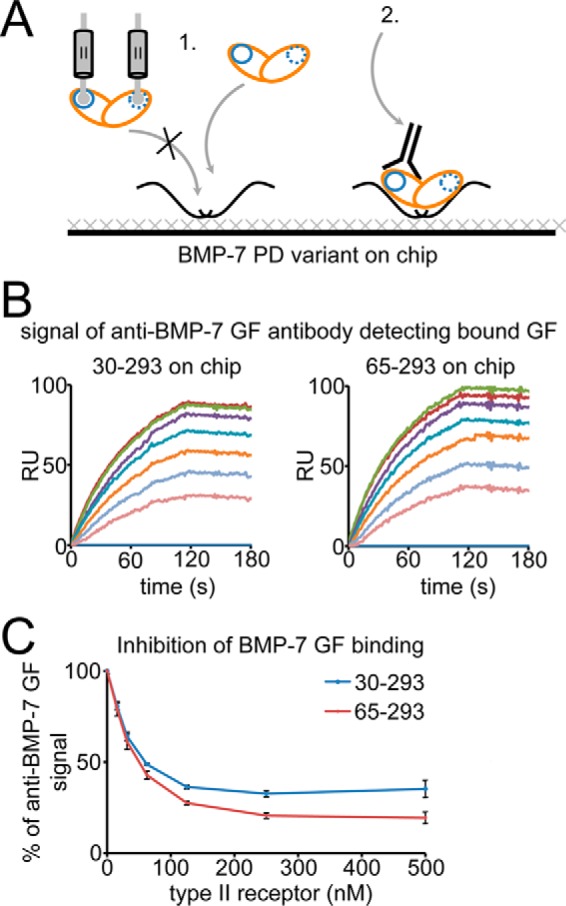
**The ^65^PHRP^68^ motif within the N-terminal region of the BMP-7 PD is required for competition with the BMP type II receptor for GF binding.** *A*, scheme of the experimental set-up. Full-length BMP-7 PD (residues 30–292) and the N-terminal truncation variant 65–292 were immobilized, and 100 nm BMP-7 GF was injected in the presence of 0–500 nm BMPRII receptor extracellular domain onto the chip first, followed by a second injection of 100 nm mAb anti-BMP-7 GF antibody to detect bound GF (all injections were in HBS-EP buffer). *B*, sensorgrams of 100 nm injected mAb anti-BMP-7 GF antibody to detect bound GF. *C*, increasing amounts of receptor resulted in comparable inhibition of GF binding to both immobilized PD variants, suggesting that the presence of the ^65^PHRP^68^ motif in 65–292 is responsible for PD competition with the type II receptor for the same binding site on the GF. *Error bars*, S.D. from three independent experiments. The schematic shows GF (*orange*) and type II receptor (*blue*) binding sites.

To test whether the ^65^PHRP^68^ motif on the BMP-7 PD is required for type II receptor competition, we carried out SPR binding studies with construct 65–292 immobilized on a chip and the GF flown over in the presence of increasing amounts of BMPRII (Fig. 7). The presence of bound BMP-7 GF was monitored by the signal of a subsequent injection of monoclonal anti-BMP-7 GF antibody (Fig. 7). In this experiment, 65–292 showed competition with the BMPRII comparable with that of the full-length BMP-7 PD(30–292), indicating that the ^65^PHRP^68^ motif but not α1 or the Ile^58^-Leu^59^-Leu^62^-Leu^64^ motif (35), which is not present in 65–292, are required to compete for type II receptor binding to the GF.

##### Interaction of Fibrillin-1 with the BMP-7 Complex Results in a Conformational Change Conferring Latency to the Growth Factor

**FIGURE 8.**
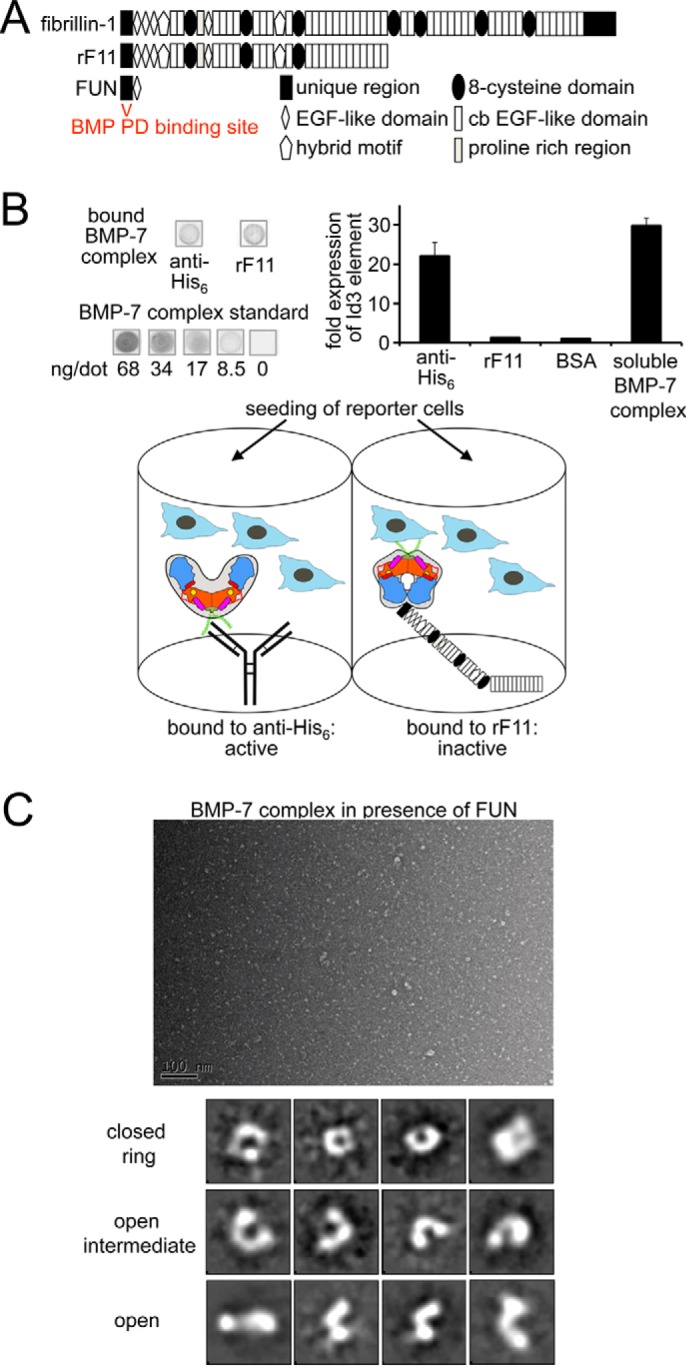
**Binding to fibrillin-1 induces a conformational change of the BMP-7 complex, resulting in GF inhibition.** *A*, domain structure of fibrillin-1 and used variants. *B*, BMP activity assay with BMP-7 complex captured via PD interactions, mAb against the N-terminal His_6_ tag, or the N-terminal half of fibrillin-1 (rF11). C2C12 cells were seeded onto immobilized BMP-7 complex, and *Id3* expression was measured as a read-out for BMP activity. Shown is dot blotting analysis of stripped BMP-7 complex by comparison with a diluted series of dots containing BMP-7 complex at known concentrations. The schematic depicts the different ways BMP-7 PD-GF complex is presented to the reporter cells. Antibody capture of the N-terminally placed His_6_ tag on the PD (*green*) does not affect bioactivity; however, binding of fibrillin-1 within the PD (*blue*) induces a conformational change into a ring shape that confers latency. *Orange*, GF dimer; *yellow circle*, type II receptor binding site; *green*, N-terminal His_6_ tag; *magenta*, α1-helix; *red*, α2-helix; *red*, stretch connecting α1- and α2-helix containing the ^65^PHRP^68^ motif; *light blue*, C-terminal portion of BMP-7 PD. *C*, dialyzing the small fibrillin-1 N-terminal fragment FUN to BMP-7 complex resulted in the formation of ring shapes and open intermediates that were absent in the BMP-7 complex-only sample (Fig. 2*A*). Shown are a representative TEM electron micrograph (*scale bar*, 100 nm) and 12 from 100 class averages of 11,000 particles (*box size*, 28 × 28 nm). The small fibrillin-1 fragment FUN was not distinguishable from the background.

To address the question of whether PD binding to ECM scaffolds, such as fibrillin-1 microfibrils, renders the BMP-7 GF inactive, we captured the BMP-7 complex via the immobilized N-terminal half of fibrillin-1 and tested its bioactivity (Fig. 8). A monoclonal antibody directed against the N-terminal His_6_ tag of the PD served as a control capture molecule. To quantitate and compare the immobilization efficiency via the two different capture approaches, immobilized BMP-7 complex was stripped and quantitated by comparison with standards of known BMP-7 complex concentrations in a dot blotting immunoassay (Fig. 8*B*). Both capture approaches resulted in immobilization of quantitative amounts of BMP-7 complex. In order to measure bioactivity, C2C12 mouse myoblast cells were seeded on immobilized complexes for 5 h, followed by mRNA isolation and measurement of mRNA expression levels of *Id3*, a BMP-responsive element, by real-time quantitative PCR. Coupling of 2 ng/well of BMP-7 complex via the His_6_ tag resulted in 22-fold induction of *Id3*, whereas stimulation with 30 ng/well of BMP-7 complex in solution resulted in 30-fold *Id3* induction (Fig. 8*B*). However, coupling the BMP-7 complex via fibrillin-1 to the dish completely inhibited its bioactivity. These data suggest that binding to fibrillin-1 denies BMP receptor access to the GF. To analyze whether BMP-7 inactivation upon binding to fibrillin-1 results in a conformational change of the complex, we employed single particle TEM. Titration experiments with a short fibrillin-1 fragment containing the N-terminal unique domain (FUN) and the adjacent first EGF-like domain showed that indeed at a 1:4 molar ratio of BMP-7 complex to fibrillin-1, we observe the presence of ring-shaped BMP-7 molecules (Fig. 8*C*). In BMP-7 complex control samples, molecules of this shape were absent (Fig. 2*A*). The small fibrillin-1 fragment was too small to be imaged on its own, and it was not possible to tell whether it was an integral part of the ring shape in these experiments (Fig. 8*C*). This clearly suggests that upon fibrillin-1 binding to the PD of the BMP-7 complex, a conformational change is induced that renders the complex inactive.

##### Interaction Studies to Identify the Fibrillin-1 Binding Motif within the BMP-7 Prodomain

**FIGURE 9.**
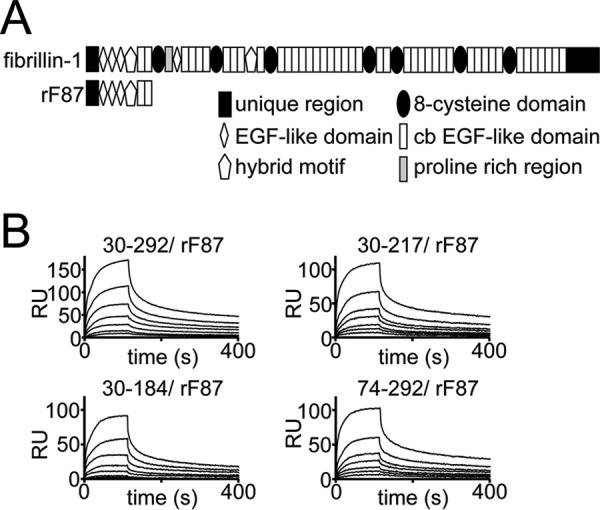
**Interaction studies to identify the fibrillin-1 binding motif within the BMP-7 PD.** *A*, domain structure of the N-terminal fibrillin-1 fragment used in the interaction study. *B*, sensorgrams of SPR binding studies suggesting that the fibrillin-1 binding domain within the BMP-7 PD resides within residues Gly^74^–Phe^185^. 0–80 nm rF87, containing the N-terminal unique domain, the first three EGF-like domains, the first hybrid motif, and the first two calcium-binding EGF- like domains of fibrillin-1, was injected onto immobilized BMP-7 PD variants. All injections were performed in HBS-EP buffer.

Based on the sequence homology between PDs of TGF-β-1 and BMP-7 and similar ability to bind to ECM molecules (LAP binds to LTBPs, and BMP-7 PD binds to fibrillin-1 and -2), it was predicted that the N-terminal Arg^51^-Glu^53^-Arg^56^-Ser^60^ motif in the BMP-7 PD contains the interaction site with fibrillin-1 (35). To test this hypothesis, we performed an interaction screen with our BMP-7 PD truncation variants and the N-terminal end of fibrillin-1 (Fig. 9*A*). We found that variant 74–292 binds with the same affinity to fibrillin-1 as the full-length PD (Fig. 9*B* and Table 3). Also, C-terminal truncation of the last 75–108 residues did not change the ability to bind fibrillin-1 in our SPR binding studies, suggesting that the fibrillin-1 binding site resides within Gly^74^–Arg^184^ rather than in the predicted Arg^51^-Glu^53^-Arg^56^-Ser^60^ motif.

## Discussion

Since the discovery of BMPs as pluripotent cytokines extractable from bone matrix under denaturing conditions, it has been speculated how BMPs are targeted and presented by the ECM (36, 37). Our previous work identified highly specific interactions between the PDs of BMPs and the extracellular glycoproteins fibrillin-1 and -2 (10, 11). Abundant extracellular co-immunostaining of BMPs and fibrillin-1 suggested that fibrillin-1 microfibrils serve as storage platforms for these GFs (20, 38). However, until now, it remained unknown whether fibrillin-1-bound BMPs are bioactive or latent. This information is highly relevant for a better understanding of disease mechanisms driven by dysregulated GF activity as present in connective tissue disorders characterized by fibrillin-1 deficiency (39, 40). Among matrix-bound BMPs, BMP-7 is probably the best investigated example. It is secreted as a very stable PD-GF complex, which can only be sufficiently separated into its components under strong denaturing conditions, such as 8 m urea (20).

However, dialysis of the BMP-7 complex together with BMP type II receptor ectodomains in physiological buffers resulted in concentration-dependent displacement of the BMP-7 PD from the GF (13). This surprising behavior provided an explanation for why BMP-7 GF and BMP-7 complex show the same bioactivity when added to reporter cells (13). Together, these findings contradicted information previously gathered about PD-GF complexes of TGF-β-1 and GDF-8 (myostatin), where the PDs serve as potent inhibitors of their cognate GFs (41, 42). However, our working hypothesis was that BMP complexes may become inactivated upon targeting to fibrillin-1 microfibrils, from which they may be utilized by cells when needed through specific, yet unknown, activation mechanisms (9, 10, 13).

**FIGURE 10.**
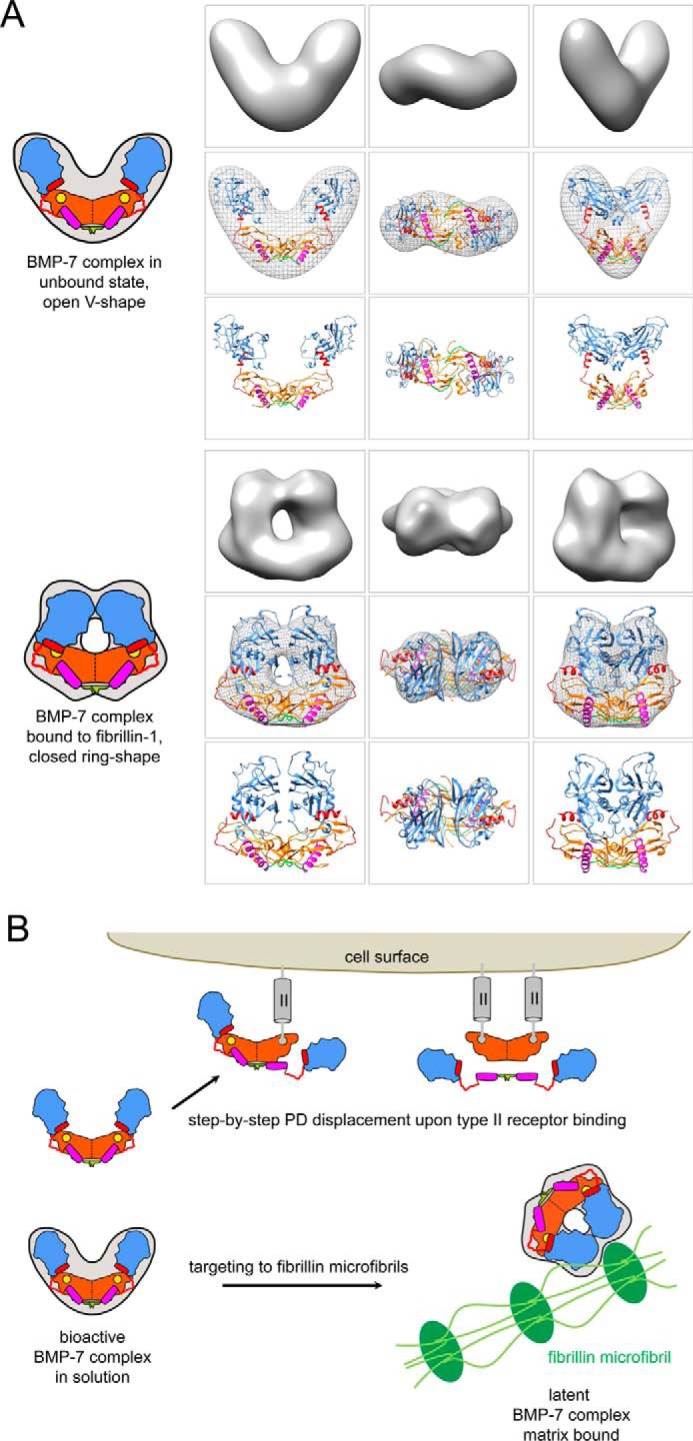
**Homology models of the BMP-7 complex and model of extracellular control of BMP GF activity via PD interactions with fibrillin-1 microfibrils.** *A* (*top*), in the unbound, bioactive state, the BMP-7 complex adopts an open V-like shape. In this conformation, the PDs are in contact with each other via the first 18 N-terminal residues (*green*). The GF shows an extended, open conformation similar to the TGF-β-1 GF in the SLC (34), which enables positioning of the α1-helix (*purple rod*) of the PD within a pocket of the GF. The PD contains a ^65^PHRP^68^ motif (*red hinge*) located between the α1- and α2-helix (*red rod*), which serves as an important “molecular clamp” for maintaining interaction with the GF and is therefore required for proper PD competition with type II receptor binding. In this conformation, the α2-helix is not occupying the type II receptor binding site on the GF. *Bottom*, upon binding to fibrillin-1, the BMP-7 complex undergoes a conformational change. In this latent, closed conformation, the two PD arms may interact with each other via unmasked C-terminal self-interaction epitopes, which in turn facilitate the ring closure. In the closed ring shape conformation, the α2 occupies the type II receptor binding site, which confers latency to the GF. *B*, in solution, binding of type II receptors to the GF moiety of the BMP-7 complex results in displacement of the PDs as a dimer. The PDs remain tethered to each other via their N-terminal self-interaction epitopes (*green*). Binding to fibrillin-1 microfibrils (*green*) induces a conformational change within the PD that enables a closed ring-shaped conformation of the BMP-7 complex, rendering the GF latent. Homology models of the BMP-7 complex in its open and closed forms were generated using the structure of the TGF-β-1 SLC (34) and fitted into the shapes determined by TEM. For the open BMP-7 form, the model is fitted in the electron density map from EM, and for the closed form the model is shown as electron density rendered at 20 Å resolution. *Orange*, GF dimer; *yellow circle*, type II receptor binding site; *green*, N-terminal self-interaction epitope; *magenta*, α1-helix; *red*, α2-helix; *red*, stretch connecting α1- and α2-helix containing the ^65^PHRP^68^ motif; *light blue*, C-terminal portion of BMP-7 PD.

Here, our biochemical and structural investigations present the first evidence that BMP activity is specifically controlled by fibrillin-1-PD interactions within the extracellular space. In our model, the untargeted BMP-7 complex assumes an open V-shape in which the two non-covalently associated PDs form a dimer via N-terminal residues (Fig. 10*A*). The GF shows an extended, open conformation similar to the TGF-β-1 GF in the SLC (34), which enables positioning of the α1-helix of the PD within a pocket of the GF. Our biochemical data suggest that this interaction is important for complex stability. The PD contains a ^65^PHRP^68^ motif located between the α1- and α2-helix, which serves as an important “molecular clamp” for maintaining interaction with the GF and is therefore required for proper PD competition with type II receptor binding. Because PD truncation variant 74–292 containing the α2-helix does not interact with the GF, we predict that in the open bioactive V-shape conformation, the α2-helix *per se* does not engage in measurable interaction with the type II receptor site. In solution, upon type II receptor binding to the BMP-7 complex, the two PDs are displaced from the GF in a step-by-step process but remain bound to each other via their N-terminal self-interaction epitopes (Fig. 10*B*). Thereby, the GF also undergoes a conformational rearrangement, rendering it from an extended/open to a closed conformation. During this process, the α1-helix of the BMP-7 PD is displaced from its binding pocket and replaced by the α2-helix of the GF. Binding of fibrillin-1 to the BMP-7 PD induces a conformational change within the PD that enables the formation of a closed ring-shaped conformation of the BMP-7 complex in which the α2-helix blocks the type II receptor binding site on the GF, rendering the GF latent (Fig. 10*B*).

Previous mutational analysis of TGF-β-1 PD residues identified a region within the first α-helix near the N terminus that participates in both GF binding and interactions with the extracellular matrix molecule LTBP-1 (35). Within this region, four hydrophobic residues (Ile^53^-Leu^54^-Leu^57^-Leu^59^) form critical interactions with the GF, whereas four charged residues (Arg^45^-Arg^50^-Lys^56^-Arg^58^) protrude from the helix to interact with LTBP-1 (35). These two motifs are somewhat conserved within the TGF-β family, which raises the possibility of a general mechanism (35). However, our truncation analysis revealed that upon truncation of the corresponding region in the BMP-7 PD, binding to the GF was only reduced by 35%, and a truncation variant lacking this region performed equally as the full-length PD in competition studies with the BMP type II receptor (Fig. 7). These data indicate the existence of additional GF binding sites within the BMP-7 PD. Here, we identify for the first time a conserved ^65^PHRP^68^ motif within the BMP-5, -6, -7 subgroup, which, when deleted, results in complete ablation of GF binding. We therefore propose that both regions are required in tandem for proper PD positioning and binding to the GF. Furthermore, our CD data indicate significant differences between the N termini of LAP and the BMP-7 PD. In BMP-7, the α1 is considerably shorter and preceded by a flexible, unordered stretch of N-terminal residues enabling PD self-interaction. Together, we conclude that all three binding motifs (residues Asp^30^–Arg^47^ as N-terminal self-interaction motif, ^65^PHRP^68^ as molecular clamp, and the predicted Ile^58^-Leu^59^-Leu^62^-Leu^64^ motif) contribute to the extraordinary stability of the BMP-7 complex in a signaling-competent open V-shape conformation.

Based on sequence alignments of TGF-β superfamily PDs, it was predicted that a stretch containing conserved hydrophobic residues (Trp^223^, Phe^226^–Ile^228^, Trp^235^-Val^236^, Leu^245^, and Leu^247^) is important for BMP-7 PD dimer formation (35). Our SPR protein-protein interaction studies using BMP-7 PD truncation variants showed that N-terminal truncation variants 74–292, 116–292, and 185–292 did not interact with full-length BMP-7 PD, whereas PD variants 218–292 and 241–292 covering the C-terminal end showed specific interaction. This suggests that in the presence of Phe^185^–Ala^217^, which contains, according to our CD data, the α3-helix, the C-terminal PD-PD interaction site is cryptic. However, the α3-helix itself does not participate in PD self-binding because 166–217 does not interact with the full-length PD. We therefore propose that upon fibrillin-1 binding to the BMP-7 complex, the PD undergoes a conformational change resulting in unmasking of the C-terminal PD self-interaction site, which in turn facilitates the ring closure. Our further binding studies show that variant 74–292 binds to fibrillin-1 with the same affinity as the full-length PD (Fig. 9*B* and Table 3). These data indicate that the fibrillin-1 binding site resides within Gly^74^–Arg^184^ rather than in the predicted Arg^51^-Glu^53^-Arg^56^-Ser^60^ motif (35).

Currently, we are fine mapping the fibrillin-1 binding site within the BMP-7 PD and also narrowing down the critical residues required for BMP PD binding within the fibrillin-1 N-terminal unique domain (FUN). Results from these studies may allow us to understand which surface PD residues are targeted by fibrillin-1 and how this results in the conformational rearrangement of the entire complex. Our single particle TEM analysis demonstrated that dialyzing a small fibrillin-1 fragment containing FUN to the BMP-7 complex resulted in the formation of ring shapes that were absent in the BMP-7 complex-only sample. Because FUN is very small, it was not distinguishable from the background (Fig. 8*C*). Therefore, we cannot speculate whether FUN as a whole or a portion of it may serve as an integral part of the ring-shaped structure or not. Because the open complex conformations detected in both the presence and absence of FUN appear very similar at this resolution, we conclude that when FUN is not bound, the BMP-7 complex remains in the open conformation.

Our proposed model of extracellular control of BMP GF activity via PD interactions with fibrillin-1 microfibrils may generally apply to matrix-bound BMP complexes that are bioactive when not targeted. This implies that in the absence or presence of only little matrix, as during early embryogenesis, BMP complexes need to be controlled by specific BMP inhibitors; however, during later embryonic stages or in postnatal life, when matrix becomes more abundant, complexes are regulated through their specific interactions with the ECM. However, there are also BMP complexes that may not be matrix-bound in which the PD serves a different function. For instance, BMP-9, which is specifically produced in liver and bioactive as a complex, circles within the bloodstream and is thereby required for early postnatal vascularization (12, 43, 44). Further, it was reported that the BMP-9 PD enables its cognate GF to exclusively select certain type II receptors for binding, whereas others are only weakly bound (33). This is accomplished by a specialized surface interface of the α2-helix with the GF (33). In contrast, the BMP-7 complex binds with the same affinities to type II receptors as the GF (13). However, in a recently published study, also no differences in *K_D_* values were measured for the BMP-9 complex and the free GF when recombinant type II receptor ectodomains (BMPRII, ActRIIA, and ActRIIB) were utilized in SPR-binding experiments (45). The discrepancy between the two studies may be explained by the different experimental set-up of the binding experiments. Similar to the results obtained with immobilized BMP-9 complex (33), we also observed that immobilization of the BMP-7 complex either through direct coating to ELISA plates or covalent cross-linking to SPR sensor chips ablated type II receptor binding (13). This suggests that BMP complex immobilization severely interferes with the PD displacement mechanism (13). However, by measuring the same interactions in reverse orientation (receptors immobilized and BMP-7 GF and complex in solution), no binding differences for complex and GF were observed, as was also recently shown for BMP-9 (13, 45). This suggests that inhibition of BMP complex bioactivity may be achieved by any means of interfering with the PD displacement mechanism, such as random tethering of the PD to solid phase during immobilization procedures.

It is currently not known whether the BMP-9 complex can be targeted to the ECM; however, similar to BMP-7, it also adopts an open V-shape (33). Furthermore, computational modeling suggested that the BMP-9 complex may also assume a closed SLC-like conformation in which the PD flips upside down and growth factor contact is turned over to the α1-helix (33). However, despite the similarity in shape, our biochemical data suggest that the PD-GF interaction requirements for BMP-7 and BMP-9 significantly differ from each other and that the conformational arrangement of the two PDs to the GF in the BMP-7 complex shows more similarity to the TGF-β1 SLC. First, both the PD-GF interaction and the PD-PD self-interaction are mediated by N-terminal residues of the BMP-7 PD. In contrast to that, the structure of the BMP-9 PD-GF complex revealed that the α5-helix residing within the C terminus of the BMP-9 PD is the main structural motif interacting with the GF (33). Interestingly, a corresponding α5-helix in the BMP-7 PD does not exist and is also absent from most other BMP PDs (33). Second, although truncation of the α1-helix in the BMP-7 PD significantly reduced BMP-7 complex stability (Fig. 6*C*), the corresponding region in the BMP-9 PD does not seem to contribute to the overall BMP-9 complex conformation. Although the N-terminal region of the BMP-9 PD composed of the first 61 residues contains also a putative α1-helix and a hydrophobic Leu^67^-Leu^70^-Leu^72^ motif corresponding to the one present in the TGF-β1 and BMP-7 PDs, this region could not be determined within the structure of the BMP-9 complex due to an observed low electron density (33). This suggests that the first N-terminal stretch of 61 residues in the BMP-9 PD is very flexible and is not required for correct positioning of the two PDs to the GF, making an N-terminal self-interaction of the two BMP-9 PDs rather unlikely. Furthermore, the crystal structure of the BMP-9 complex revealed that the two PDs do not make contact via C-terminal residues; however, a self-interaction affinity with isolated BMP-9 PDs could still be measured and was determined to be in the micromolar range (33). In contrast, our data show strong BMP-7 PD self-interaction with an affinity of 3 nm, which is higher than the PD-GF affinity of 20 nm (13) and may explain the extraordinary stability of the BMP-7 complex and, further, how the PDs can stay attached to each other once the PD-GF interaction is released upon type II receptor binding (13). Third, in the TGF-β1 SLC complex structure, a “straitjacket” composed of the PD α1-helix and latency lasso encircles the GF on the side opposite the arm domain (34). Within the corresponding straitjacket region in BMP-7 PD resides the newly identified conserved ^65^PHRP^68^ motif, which we found to be important for GF binding. Moreover, this motif is also present in the TGF-β1 PD but not in the BMP-9 PD. Fourth, the crystal structures of the BMP-9 GF alone (12), the BMP-9 GF bound to type I and the type II receptors (46), and the BMP-9 GF within the BMP-9 PD-GF complex (33) all show perfect overlay, suggesting that, unlike the TGF-β GF, the BMP-9 GF does not undergo a conformational change during receptor binding and PD displacement. In our model, we propose that the α1-helix of the BMP-7 PD interacts with the GF by residing in a pocket that becomes available when the growth factor has a more open, extended conformation, as is the case for the TGF-β1 GF within the SLC. The BMP-9 GF does not have such a pocket, neither alone nor in the complexed form together with the PD.

Our gathered structural and biochemical data suggest that the BMP-7 complex is more stable than the BMP-9 complex. The BMP-7 PD-GF affinity seems to be significantly higher when compared with BMP-9 (*K_D_* = 20 nm (13) *versus* 0.8 μm (33)) and, in contrast to the BMP-9, also robust to the presence of up to 1 m urea or pH reduction down to 4.5 (Fig. 5*B* and Table 3).

Taken together, the differences between BMP-7 and -9 complex structures may be general characteristics of soluble *versus* matrix-bound PD-GF complexes and reflect their individual functional requirements. Because the BMP-9 complex appears to be less stable compared with BMP-7, it has been speculated that a regulatory role of its PD is not very likely (46) and that the main function of the PD is making the growth factor larger in size, which prevents it from renal filtration (46). On the other hand, BMP-7 is found to be stored in tissues and must underlie tight conformational regulation to prevent harmful aberrant signaling events.

However, future investigations are needed to reveal the molecular requirements of TGF-β superfamily complexes with regard to their specific biological function. For instance, we previously showed that the PD of BMP-10 is a specific and potent inhibitor of its cognate GF (13). However, a recent report demonstrated that this control is cell type-specific, suggesting the involvement of additional mediators, such as co-receptors, in the signaling mechanism of BMP PD-GF complexes (47). Because BMP-10 is the closest relative to BMP-9 and contains an α5-helix in its PD, it will be interesting to investigate whether the BMP-10 complex adopts an open V- or closed ring shape conformation in the unbound state.

## Author Contributions

G. S. conceived and coordinated the study and wrote the paper. A. P. W. designed, performed, and analyzed the experiments shown in Figs. 1, 4–7, 8 (*A* and *B*), 9, and 10. H. T., R. F. C., and C. B. designed, performed, and analyzed the experiments shown in Figs. 2, 3, 8*C*, and 10*A*. All authors reviewed the results and approved the final version of the manuscript.

## Footnotes

2 The abbreviations used are:

BMP: bone morphogenetic protein
ECM: extracellular matrix
FUN: fibrillin-1 unique N-terminal domain
FMF: fibrillin microfibrils
GF: growth factor
LAP: latency-associated peptide
LTBP: latent TGF-β-binding protein
PD: prodomain
rF: recombinant fibrillin
RU: resonance units
SLC: small latent complex
SPR: surface plasmon resonance
TEM: transmission electron microscopy
SAXS: small-angle x-ray scattering.
